# Fulminant ACTH decrease following diabetic ketoacidosis induced by immune checkpoint inhibitor combination therapy with nivolumab and ipilimumab: A case report

**DOI:** 10.1097/MD.0000000000036664

**Published:** 2023-12-22

**Authors:** Hiroshi Iesaka, Hiraku Kameda, Aika Miya, Hiroshi Nomoto, Kyu Yong Cho, Akinobu Nakamura, Takashige Abe, Nobuo Shinohara, Tatsuya Atsumi

**Affiliations:** a Department of Rheumatology, Endocrinology and Nephrology, Faculty of Medicine and Graduate School of Medicine, Hokkaido University, Sapporo, Japan; b Department of Renal and Genitourinary Surgery, Graduate School of Medicine, Hokkaido University, Sapporo, Japan.

**Keywords:** adrenocorticotropic hormone (ACTH), fulminant type 1 diabetes, hypophysitis, immune checkpoint inhibitor, secondary adrenal insufficiency

## Abstract

**Rationale:**

The increasing use of immune checkpoint inhibitors (ICIs) for treating malignant tumors result in the concomitant rise of immune-related adverse events (irAEs). This case report may provide useful insight to understanding the etiology of ICI-induced hypophysitis, a severe irAE leading to potentially fatal secondary adrenal insufficiency.

**Patient concerns:**

An 81-year-old Japanese man was hospitalized for diabetic ketoacidosis following 4 courses of ICI combination therapy with nivolumab and ipilimumab for metastatic renal cell carcinoma.

**Diagnosis:**

Insulin secretion was depleted, leading to diagnosis of fulminant type 1 diabetes. Adrenocorticotropic hormone (ACTH) and cortisol levels were very high (60.8 pmol/L and 1575 nmol/L, respectively) upon admission. ACTH and cortisol returned to normal ranges on the 2nd day. On the 8th day, an ACTH loading test showed intact cortisol response (peak value 519 nmol/L). However, on the 14th day, there was a sharp decrease in ACTH and cortisol levels (10.5 pmol/L and 47 nmol/L, respectively) accompanied by fatigue and a drop in blood pressure to 97/63 mm Hg. As secondary adrenal insufficiency was suspected, hydrocortisone replacement was initiated. An ACTH loading test on the 17th day revealed low cortisol peak (peak value 232 nmol/L), indicating sudden disruption of adrenal function. Magnetic resonance imaging showed no abnormal findings and there was no other pituitary hormone deficiency. These findings, along with the patient clinical course, suggest that secondary adrenal insufficiency was caused by acute ACTH producing cell destruction as an irAE associated with ICI therapy.

**Interventions:**

The patient hyperglycemia and ketoacidosis were treated using extracellular fluid and insulin therapy. After development of adrenal insufficiency, hydrocortisone 20 mg was started, and the patient symptoms improved.

**Outcomes:**

He was continued on insulin therapy, hydrocortisone, and reinitiated nivolumab.

**Lessons:**

This case provides a detailed course of the fulminant onset of ACTH deficiency during ICI administration, emphasizing the importance of close monitoring.

## 1. Introduction

The use of immune checkpoint inhibitors (ICIs) is increasing because of their broad indication for treatment of malignant tumors, resulting in the concomitant rise of immune-related adverse events (irAEs).^[1]^ Several cases with plural irAEs have been previously reported, although the clinical course and underlying mechanisms are still unclear.^[2–4]^ Hypophysitis is one of the critical irAEs, which develops acute secondary adrenal insufficiency, triggering to a fatal outcome. We previously reported nivolumab-induced hypophysitis leading to secondary adrenal insufficiency after transient adrenocorticotropic hormone (ACTH) elevation. This case report suggested that increased ACTH is an early indicator of the hypophysitis onset.^[5]^ We here present the case of a patient with renal cell cancer who received nivolumab and ipilimumab combination therapy and developed thyroid dysfunction initially and was then hospitalized due to fulminant hypophysitis following diabetic ketoacidosis caused by the onset of fulminant type 1 diabetes (T1D). While hospitalized, the patient also developed adrenal insufficiency caused by the hypophysitis. Since we intensively followed the course of the hypophysitis onset by monitoring ACTH, cortisol, and ACTH loading at pre- and post-onset, this case report would give useful insight to understanding the etiology of fulminant ICI-induced hypophysitis.

## 2. Case presentation

An 81-year-old Japanese man was found to have a 21 mm left renal tumor after having an abdominal computed tomography scan. The patient was introduced to a nearby hospital, where a robot-assisted laparoscopic partial nephrectomy was performed, and the tumor was diagnosed as clear cell renal cell carcinoma. 7 months after, the patient complained of left back pain and a computed tomography scan showed bone metastasis in the 12th thoracic and 5th lumbar vertebrae. Denosumab was given, followed by initiation of ICI combination therapy with nivolumab and ipilimumab 3 months after starting denosumab treatment.

At a regular visit after 2 courses of ICI combination therapy, thyrotoxicosis (thyroid-stimulating hormone [TSH] 0.02 mIU/L, free triiodothyronine 8.66 pmol/L, free thyroxine 35.9 pmol/L) without any symptoms was discovered. Based on the finding of negative TSH receptor antibody but positive anti-thyroglobulin antibody without increased vascularity on color Doppler ultrasound, the patient was thought to have developed destructive thyroiditis due to ICI combination therapy with nivolumab and ipilimumab. The thyrotoxicosis had improved in natural course without medication.

Following 4 courses of ICI combination therapy, the patient presented at the hospital with nausea, loss of appetite, and thirst lasting for 3 days. Blood tests showed hyperglycemia (plasma glucose 34.6 mmol/L, hemoglobin A1c 47 mmol/mol) and ketoacidosis, and the patient was emergently hospitalized immediately (Table 1). Serum C-peptide immunoreactivity (CPR) levels were undetectable in the fasting state and after loading with 1 mg glucagon, exhibiting insulin depletion. Anti-GAD antibody was negative. The patient developed ketoacidosis within 1 week of symptom onset, with plasma glucose > 16.0 mmol/L (288 mg/dL), hemoglobin A1c (IFCC) < 72 mmol/mol (NGSP, <8.7%), and serum CPR < 0.17 nmol/L (<0.5 ng/mL) after glucagon load. Therefore, the patient was diagnosed with fulminant T1D mellitus.^[6]^ The patient was identified as having the human leukocyte antigen (HLA) class II alleles DRB1*09:01 and DQB1*03:03, which are related to susceptibility to T1D.^[7]^ The patient hyperglycemia and ketoacidosis were managed with extracellular fluid monitoring, continuous venous insulin infusion, and multiple daily insulin injections. ICI combination therapy was stopped.

**Table 1:** Laboratory findings on admission.

Hematology			Biochemistry		
White blood cell	12.8 × 10^9^	/L	Total protein	75	g/L
Neutrophil	86.0	%	Albumin	43	g/L
Lymphocyte	7.0	%	Creatinine	99.9	µmol/L
Monocyte	7.0	%	Blood urea nitrogen	15.7	mmol/L
Hemoglobin	129	g/L	Sodium	133	mmol/L
Red blood cell	4.90 × 10^12^	/L	Potassium	5.4	mmol/L
Hematocrit	0.404	/L	Chloride	93	mmol/L
Platelet	201	/L	Aspartate aminotransferase	35	IU/L
			Alanine aminotransferase	59	IU/L
			Total cholesterol	3.1	mmol/L
			Triglyceride	0.3	mmol/L
			Postprandial plasma glucose	34.6	mmol/L
			HbA1c	47	mmol/mol
			Venous blood gas (room air)		
			pH	7.215	
			pCO_2_	30.1	mm Hg
			HCO^3-^	11.7	mmol/L
			Base excess	−14.7	mmol/L
			Anion gap	21.3	mmol/L
			Total ketone body	13.468	mmol/L
			3-hydroxybutyric acid	10.496	mmol/L
			Acetoacetic acid	2.972	mmol/L
			Human leukocyte antigen	DRB1*09:01-DQB1*03:03	

**Figure 1.**
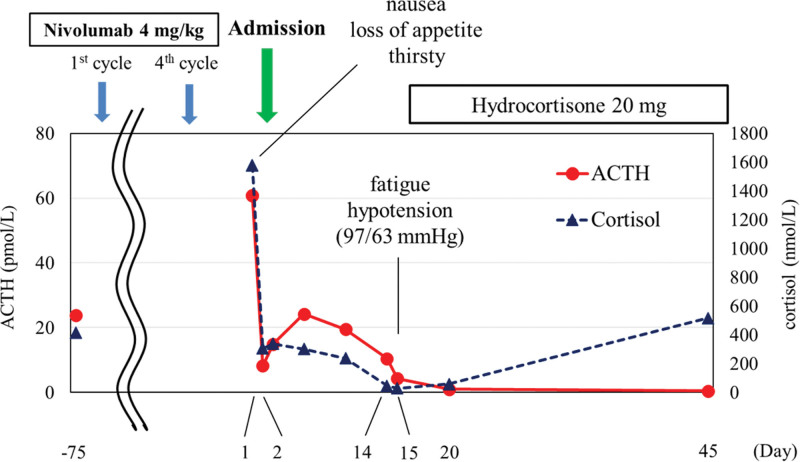
Changes in blood ACTH and cortisol levels. ACTH = adrenocorticotropic hormone.

**Figure 2.**
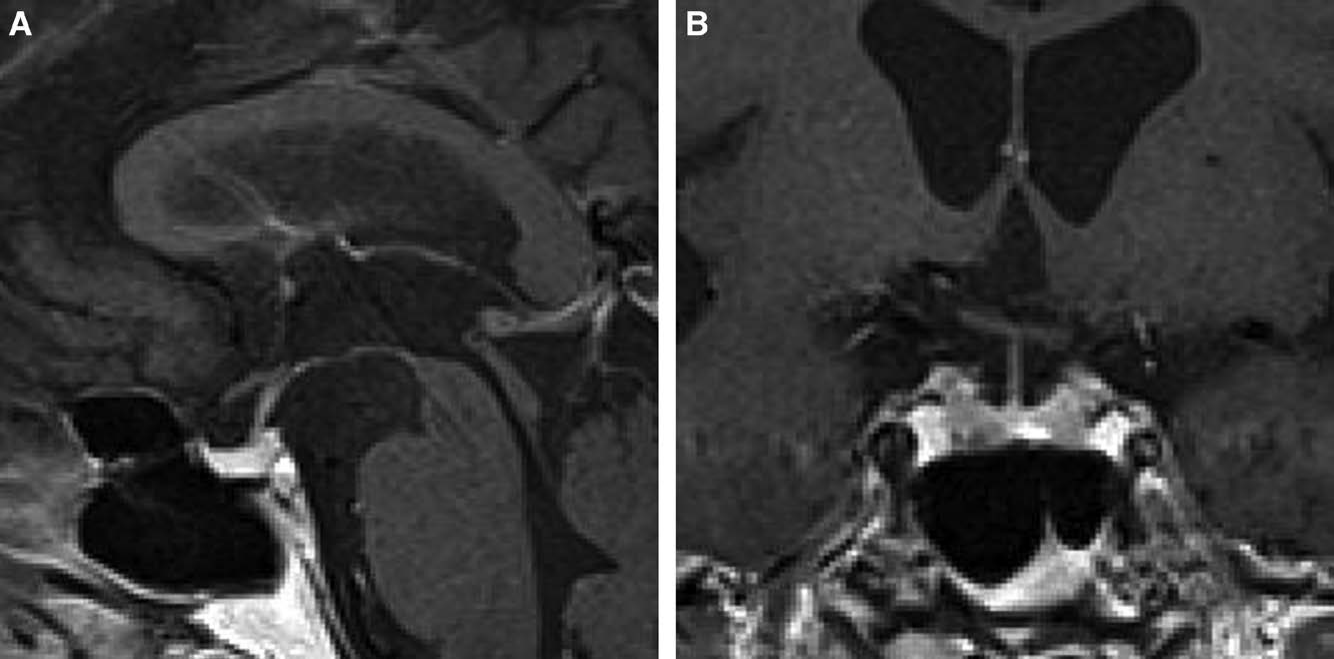
Brain magnetic resonance imaging (MRI) on d 20. (A) Sagittal gadolinium-enhanced T1-weighted MRI. (B) Coronal gadolinium-enhanced T1-weighted MRI.

**Figure 3.**
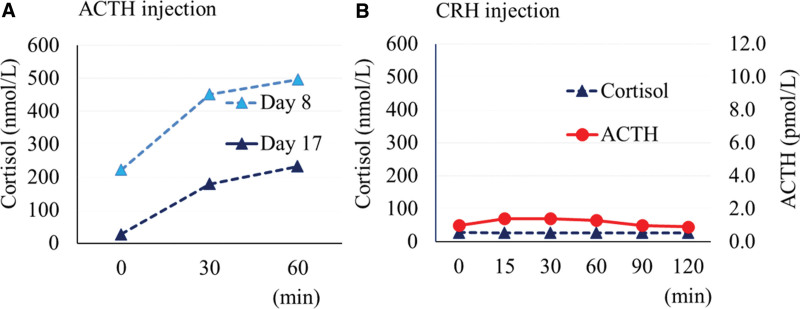
Results of hormone stimulation tests. (A) Changes in cortisol after intravenous ACTH (250 µg) injection. (B) Changes in ACTH and cortisol after intravenous CRH (0.1 mg) injection on d 21. ACTH = adrenocorticotropic hormone, CRH = corticotropin-releasing hormone.

ACTH and cortisol levels were strikingly high (60.8 pmol/L and 1575 nmol/L, respectively) on the admission day (day 1) (Table 2), which may be explained by physical stress and diabetic ketoacidosis; however, having learned from a previously reported a case of nivolumab-induced hypophysitis leading to secondary adrenal insufficiency after transient ACTH elevation ^[5]^, we frequently monitored the patient ACTH and cortisol levels. ACTH and cortisol levels returned to normal ranges (8.2 pmol/L and 303 nmol/L) on day 2, and ACTH increased again to 24.2 pmol/L on day 6, whereas cortisol level remained normal (301 nmol/L). An ACTH loading test showed intact cortisol response (peak value: 519 nmol/L) on day 8. On day 14, ACTH and cortisol levels decreased sharply to 10.5 pmol/L and 47 nmol/L in the early morning (Fig. 1), and the patient complained of fatigue and the patient blood pressure had decreased to 97/63 mm Hg. Secondary adrenal insufficiency was suspected, and hydrocortisone replacement (15 and 5 mg, in the morning and evening, respectively) was started. An ACTH loading test was performed again, and basal cortisol value was < 28 nmol/L with peak value 232 nmol/L, suggesting adrenal insufficiency. Magnetic resonance imaging (MRI) showed no abnormal findings in the patient pituitary gland on day 20 (Fig. 2). A corticotropin-releasing hormone (CRH)-thyrotropin-releasing hormone-luteinizing hormone-releasing hormone loading test on day 21 showed normal responses for prolactin, luteinizing hormone, and follicle-stimulating hormone, however, ACTH showed low response to CRH, and cortisol did not respond to CRH increased ACTH (Fig. 3). Thyrotropin response to thyrotropin-releasing hormone was reduced because of levothyroxine administration. A growth hormone releasing peptide-2 loading test showed normal response of growth hormone.

**Table 2:** Basal endocrinological data on admission.

Endocrinology		
	Value	Unit (reference value)
Adrenocorticotropic hormone	60.8	pmol/L (1.5–12.3)
Cortisol	1575	nmol/L (176–579)
Thyroid stimulating hormone	0.01	mU/L (0.38–4.31)
Free triiodothyronine	2.65	pmol/L (3.23–5.83)
Free thyroxine	18.0	pmol/L (10.6–21.0)
Anti-thyroglobulin antibody	679.74	IU/mL (0–0.2)
Anti-thyroid peroxidase antibody	<0.05	IU/mL (0–0.2)
Growth hormone	1.02	µg/L (0.11–3.90)
Insulin-like growth factor-1	7.87	nmol/L (6.4–23.6)
Prolactin	12.6	µg/L (4.1–28.9)
Luteinizing hormone	5.1	IU/L (1.8–5.2)
Follicle stimulating hormone	20.2	IU/L (2.9–8.2)
Testosterone (ECLIA)	3.16	nmol/L (6.93–26.35)

The above findings and the patient clinical course suggested secondary adrenal insufficiency arising from ICI-induced hypophysitis. Administration of 20 mg hydrocortisone was continued. During the course of hospitalization, a notable decline in thyroid hormones was observed on day 8, and as a therapeutic intervention, an initial dosage of 25 µg of levothyroxine was administered on day 9. Subsequently, the dosages were gradually escalated to 50 µg on day 15 and further increased to 75 µg on day 21, resulting in normalization of thyroid hormone levels. The patient was discharged on day 31. The monotherapy of nivolumab was started after discharge with hormone replacement. The patient hormone status was unchanged 4 months post discharge.

## 3. Discussion and conclusions

Here we report a case of ICI combination therapy-induced fulminant ACTH deficiency following thyroiditis and fulminant T1D, and we present, for the first time, the hormonal status pre- and post-onset of ACTH deficiency including ACTH loading test results. There was no other anterior pituitary hormone or arginine vasopressin deficiency observed.

Table 3 illustrates the knowledge of hypophysitis induced by ICIs. Several meta-analyses reported that the incidence rate of hypophysitis with ICI combination therapy is 7.7% to 10.5%.^[8–12]^ Barroso-Sousa^[8]^ reported that the patients who received ICI combination therapy were more likely to develop hypophysitis (odds ratio 2.2; 95%CI 1.39–3.60) than those who received ipilimumab monotherapy. Da^[12]^ reported that ICI combination therapy significantly increased the risks of hypophysitis (relative risk 3.5; 95%CI 2.07–6.07) when compared with ICI monotherapy. Furthermore, other endocrine irAEs, such as hypothyroidism (risk ratio [RR], 2.17) and hyperthyroidism (RR, 3.13), and non-endocrine irAEs, including pneumonitis (RR, 2.31), and hepatitis (RR, 2.54), occurred more frequently with ICI combination therapy than with ICI monotherapy.^[12]^ Therefore, the prevalence of developing multiple irAEs, such as in this case, is assumed to increase. The clinical features of hypopituitarism are contingent upon the specific type of ICI administered. For instance, nivolumab-induced hypophysitis frequently demonstrates no noticeable change in the hypopituitary gland on MRI,^[13]^ with isolated ACTH deficiency (IAD) being a prevalent occurrence.^[14]^ In contrast, enlarged pituitary gland appeared on MRI in 60 cases out of 61 patients with ipilimumab-induced hypophysitis,^[13]^ with multiple anterior pituitary hormones being implicated. A review of the hormone disruption patterns revealed TSH 92%, luteinizing hormone/follicle-stimulating hormone 85.7%, ACTH 74%, prolactin 64.7%^[15]^ in cases of ipilimumab-related hypophysitis. The incidence of GH deficiency was 41.9%, a comparatively low rate. Reports of diabetes insipidus remain scarce, with only 1 out of 75 cases,^[16]^ whereas Atkins^[17]^ documented a 36% rate (4 cases out of 11) of diabetes insipidus development.

**Table 3:** Hypophysitis due to combination therapy.

	Nivolumab (Nivo)	Ipilimumab (Ipi)	Combination therapy*
Incidence rate	0.3%–1.1%^[8–11]^	3.8%–5.6%^[8–11]^	7.7%–10.5%^[8–12]^ OR 2.2%–3.5%^[8,12]^ (vs monotherapy)
MRI	No change^[13]^	Enlargement of pituitary^[13]^	Variable
Hormone deficiency	Isolated ACTH deficiency^[14]^	Multiple anterior pituitary hormone deficiency^[15–17]^	Variable
mechanism	Autoimmune response against corticotrophs by ectopic ACTH expression in tumors^[16]^	Binding to CTLA-4 and activated classical pathway^[19]^	Unknown

ACTH = adrenocorticotropic hormone; CTLA-4 = cytotoxic T-lymphocyte associated antigen-4, MRI = magnetic resonance imaging, OR = odds ratio.

* Combination of two immune checkpoint inhibitors, e.g. PD-1/PD-L1 plus CTLA-4 inhibitors.

Immune cells possess the capability to recognize and prepare for the obliteration of cancer cells. However, their activity is hindered by the presence of inhibitory receptors and ligands including cytotoxic T-lymphocyte associated antigen-4 (CTLA-4), programmed cell death protein 1 (PD-1), and programmed death-ligand 1 (PD-L1). Under physiological conditions, these immune checkpoint pathways uphold self-tolerance and restrict incidental tissue damage during antimicrobial immune responses. In the context of oncology, these pathways can be manipulated to evade immune-mediated tumor destruction. Therapeutic agents such as anti-CTLA-4, anti-PD-1, anti-PD-L1 inhibit these receptors and ligands, which promote sustained regression of neoplasms by mitigating the suppression of antitumor immune responses.^[18]^ However, CTLA-4 antigens were expressed by pituitary endocrine cells of all subjects, including 6 patients and 11 unaffected controls who had not been received anti-CTLA-4 antibodies. However, expression levels varied. The binding of anti-CTLA-4 to CTLA-4 expressed in the pituitary gland stimulates macrophage phagocytosis and triggers the classical pathway, known as Type II hypersensitivity reactions.^[16]^ In addition, case of autoimmune reactions against corticotrophs triggered by ectopic ACTH expression in tumors among patients receiving PD-1 have been reported.^[19]^ T cell mediated immune destruction has been suggested.^[20]^ In this case, there was no signs of pituitary enlargement on MRI, and only ACTH was disordered, a characteristic feature observed in nivolumab-induced hypophysitis.

Three irAEs, such as thyroid disease, diabetes mellitus, and hypophysitis, developed in this case. In 7 previous reports of triple endocrine irAEs due to ICI therapy (Table 4),^[21–27]^ thyroid disease and/or T1D mellitus developed before onset of hypophysitis. In 3 cases, ICI therapies were continued or reinitiated after resolution of irAEs.^[22,24,26]^ In 1 case, treatment was switched to sunitinib, but serum creatinine increased and nivolumab was resumed.^[25]^ In these 7 cases, the symptoms of secondary adrenal insufficiency due to hypophysitis comprised refractory hypotension, nausea, fatigue, memory loss, malaise, loss of appetite, and hypoglycemia, which lack specificity. Hyponatremia and hyperkalemia were also signs of adrenal insufficiency. There was also no detailed investigation of hormones, such as pre- and post-hypophysitis onset ACTH loading tests. This case study represents the first documentation of ACTH deficiency manifesting within an extraordinarily condensed time frame, specifically within a few days, reflecting the rapid progression characteristic of fulminant T1D. We previously reported a case of hypophysitis leading to secondary adrenal insufficiency after transient ACTH elevation by nivolumab,^[5]^ and the present report showed a similar course due to ICI combination therapy, suggesting that the observed ACTH increase may be a clue to the subsequent rapid decline in ACTH.

**Table 4:** Cases that have 3 irAEs including secondary adrenal insufficiency due to ICI therapy.

Case	Age	Sex	Primary disease	Therapy	Endocrine irAEs*	Signs of secondary adrenal insufficiency	D to hypophysitis diagnosis from first administration
Sum M (2018)^[21]^	75	M	Melanoma	Nivo + Ipi	T1D → AD = hypothyroidism	Hypoglycemia, malaise	NA
Khalid M (2019)^[22]^	53	M	Malignant melanoma	Nivo + Ipi	primary hypothyroidism → IAD → T1D	Refractory hypotension	6 mo
Lanzolla G (2019)^[23]^	60	M	Lung adeno-carcinoma	Atezo	T1D → Addison disease = hypophysitis	Hyperkalemia and hyponatremia	12 wk
Takata M (2022)^[24]^	59	M	Malignant melanoma	Nivo	Thyroiditis → IAD → acute T1D	Nausea	14 wk
Hino C (2022)^[25]^	45	M	Sarcomatoid renal cell carcinoma	Nivo + Ipi	Thyroiditis → T1D → AD	Fatigue and memory loss	244 d
Ishiguro A (2022)^[26]^	67	M	Malignant melanoma	Nivo	T1D = thyroiditis → IAD	Malaise and loss of appetite	57 d
Luo J (2022)^[27]^	76	M	Non-small cell lung cancer	Pem	Hypothyroidism → T1D → AD	Hyponatremia	14 mo
This case	81	M	Renal cell carcinoma	Nivo + Ipi	Thyroiditis → fulminant T1D → IAD	Hypotension and fatigue	89 d

Atezo = Atezolizumab, ICI = immune checkpoint inhibitor, Ipi = ipilimumab, Nivo = nivolumab, Pem = Pembrolizumab, T1D = type 1 diabetes.

* Endocrine irAEs are listed in order of onset.

Table 5 shows cases reporting hypophysitis and T1D.^[3,21–32]^ ICIs are anti-PD-1 monotherapy, anti-PD-L1 monotherapy, and combination therapy of anti-PD-1/anti-PD-L1 and anti-CTLA-4. No cases with hypophysitis and T1D due to ipilimumab monotherapy were reported. Three previous cases reported the presence of anti-glutamic acid decarboxylase antibody. T1D susceptibility-related HLA haplotypes, DRB1*08:02-DQB*03:02 and DRB1*04-DQB1*03 were reported in 2 separate cases, but no other reports of HLA alleles related to T1D susceptibility were found. Kobayashi et al^[33]^ showed that some HLA alleles are often positive in hypophysitis due to ICIs. Risk-related HLA alleles are distinct, and they overlap between IAD and panhypopituitarism. The prevalence of *HLA-Cw12, HLA-DR15, HLA-DQ7*, and *HLA-DPw9* was significantly higher in patients with IAD, whereas that of *HLA-Cw12* and *HLA-DR15* was significantly higher in patients with panhypopituitarism compared to controls. However, none of the reported HLA alleles overlapped with T1D. In the present case, the patient was negative for all autoimmune islet antibodies and the patient had a T1D risk-related HLA allele. The involvement of activated T cells in irAEs such as T1D^[34]^ and hypophysitis^[16]^ has been suggested as a mechanism, and HLA polymorphisms would play an important role in the development.^[30,33]^

**Table 5:** Cases that have T1D and hypophysitis due to ICI therapy.

Case	Age	Sex	Primary disease	Therapy	A term to hypophysitis diagnosis from T1D*	Hormone disorder	Enlarged pituitary by MRI	HbA1c (mmol/mol)	CPR (nmol/L)	Autoantibody	HLA†
Okahata S (2019)^[3]^	52	F	Breast cancer	Nivo	−1 mo	IAD	no	62	0.10	GAD-/IA-2-/IAA-	DRB1*14:05,14:06 DQB1*03:01,03:03
Sum M (2018)^[21]^	75	M	Melanoma	Nivo +Ipi	NA (T1D → hypophysitis)	AD	enlarged	60	0.10	GAD+/islet-	NA
Khalid M (2019)^[22]^	53	M	Malignant melanoma	Nivo +Ipi	−18 mo	IAD	no	NA	un- detectable	GAD+	NA
Lanzolla G (2019)^[23]^	60	M	Lung adeno-carcinoma	Atezo	1 wk	AD	no	NA	un- detectable	pituitary+ /21-hydroxylase+ /GAD-/IA-2-	DRB1*04- DQB1*03
Takata M (2022)^[24]^	59	M	Malignant melanoma	Nivo	−22 wk	IAD	NA	58	0.18	NA	NA
Hino C (2022)^[25]^	45	M	Sarcomatoid renal cell carcinoma	Nivo +Ipi	73 d	AD	NA	60	0.17	GAD-/IAA-	NA
Ishiguro A (2022)^[26]^	67	M	Malignant melanoma	Nivo	168 d	IAD	no	54	0.86	GAD+	DRB1*08:02-DQB*03:02
Luo J (2022)^[27]^	76	M	Non-small cell lung cancer	Pem	4 mo	AD	NA	27	0.01	GAD-/IA-2- /islet-	HLA-DQA1/DQB1
Marchand L (2017)^[28]^	55	M	Pulmonary pleomorphic carcinoma	Nivo	1 mo	IAD	no	66	un-detectable	GAD-/IA-2-/IAA-/islet- /Zinc 8-	NA
Porntharukchareon T (2020)^[29]^	70	M	Non-small cell lung cancer	Nivo +Ipi	simultaneous	IAD	no	48	un-detectable	GAD-/IA-2-	NA
Kikuchi F (2021)^[30]^	62	M	Renal cell carcinoma	Nivo	−3 mo	IAD	no	65	0.07	GAD-	NA
Boswell L (2021)^[31]^	51	M	Malignant melanoma	Nivo +Ipi	−75 wk	IAD	no	63	0.32	GAD-/IA-2-/IAA-	DRB*01:01, DRB1*12:01
Min Li (2022)^[32]^	45	M	Non-small-cell lung cancer	KN046‡	−2 mo	AD	no	77	<0.03	GAD-/IA-2- /islet-	NA
This case	81	M	Renal cell carcinoma	Nivo +Ipi	14 d	IAD	no	46	0.01	GAD-/IA-2-/IAA-/islet- /Zinc 8-	DRB1*09:01- DQB1*03:03

CPR = serum C-peptide, GAD = anti-glutamic acid decarboxylase antibody, HbA1c = hemoglobin A1c, IA-2 = anti-insulinoma-associated antigen-2 antibody, IAA = insulin auto-antibody, ICI = immune checkpoint inhibitor, islet = islet cell antibody, NA = not assessed, Zinc 8 = zinc transporter 8 antibody.

* Minus (−) means hypophysitis precedes T1D.

† Bold letters are HLAs susceptible to T1D.

‡Recombinant humanized PD-L1/CTLA-4 bispecific single-domain antibody Fc fusion protein injection.

Because adrenal insufficiency due to ICI therapy could be fatal, this condition should be more widely recognized by physicians with appropriate ACTH/cortisol monitoring as well as associated clinical manifestations. The present report provides a detailed course of hypophysitis, which will contribute to better care for patients receiving ICI therapy.

## Author contributions

**Conceptualization:** Hiraku Kameda.

**Investigation:** Hiroshi Iesaka, Aika Miya, Hiroshi Nomoto, Kyu Yong Cho, Akinobu Nakamura, Takashige Abe.

**Supervision:** Nobuo Shinohara, Tatsuya Atsumi.

**Writing – original draft:** Hiroshi Iesaka.

**Writing – review & editing:** Hiraku Kameda.
